# Dormant micro arteriovenous malformations lead to recurrent cerebral haemorrhage

**DOI:** 10.1186/s40064-016-2615-5

**Published:** 2016-07-11

**Authors:** Jun Cai, Hao Lin, Shaoxue Li, Zhimin Zou, Yanting Zhang, Shiwan Liu, Xin Chen, Xiaoxin Bai

**Affiliations:** Department of Neurosurgery, Hospital of Guangzhou University Mega Center, Guangdong Provincial Hospital of Chinese Medicine, Guangzhou, 510006 China; Department of Radiology, Guangdong Provincial Hospital of Chinese Medicine, Guangzhou, 510120 China

**Keywords:** Arteriovenous malformations, Cerebral haemorrhage, Digital subtraction angiography

## Abstract

**Introduction:**

Some micro arteriovenous malformations (AVMs) located in deep brain are undetectable. How to choose a proper timing to detect these AVMs remains unclear.

**Case description:**

A 21-year-old male patient was admitted to our center for intraventricular haematoma. Digital subtraction angiographies (DSAs) were performed one week and one month respectively after his haemorrhage, but no positive results were obtained. The patient was hospitalized for re-haemorrhage six years later. A micro AVM with two diffused niduses was detected and embolised three months after his re-haemorrhage. The patient recovered without any neurological deficit.

**Discussion and evaluation:**

Compressive effects of haematoma and spontaneous obliteration of AVMs might play pivotal roles in negative DSA results.

**Conclusions:**

Strategic and timely use of DSA could identify some dormant re-haemorrhagic AVMs.

**Electronic supplementary material:**

The online version of this article (doi:10.1186/s40064-016-2615-5) contains supplementary material, which is available to authorized users.

## Introduction

Rupture of brain arteriovenous malformations (AVMs) is a leading cause of cerebral haemorrhage in children and young adults, with 1.4–4.67 % of AVM patients developing cerebral haemorrhage each year (da Costa et al. 2009; Gross and Du 2013; van Beijnum et al. 2011). Some micro AVMs with unconspicuous feeding arteries and single draining veins are located in deep brain and it’s difficult to diagnose such AVMs. Moreover, these dormant and re-haemorrhagic AVMs usually lead to high mortality and morbidity.

## Case report

**Fig. 1:**
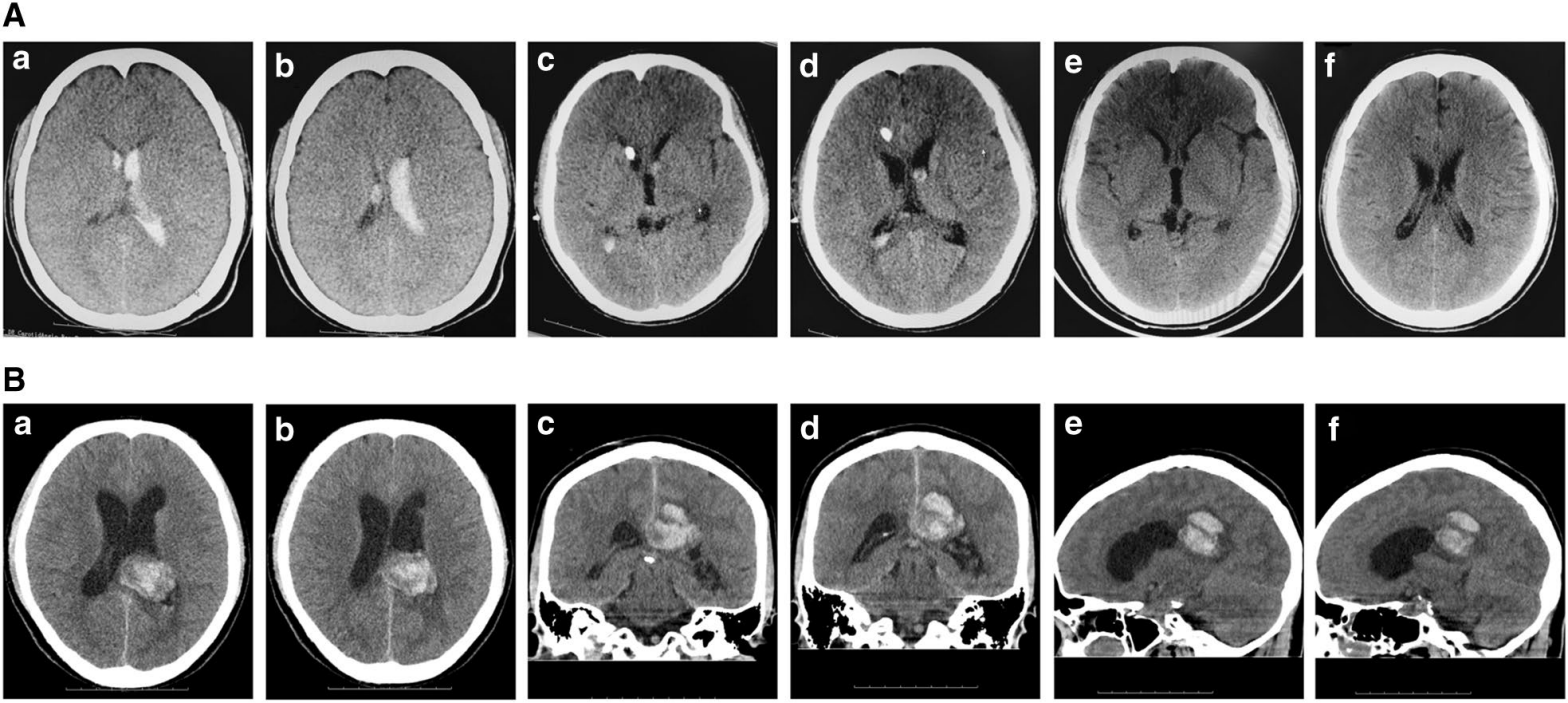
CT images showed morphologies of cerebral haemorrhage. The preceding haemorrhage was located in the ventricle (**A-** *a, b*). Ventricular drainage was employed to remove the intraventricular haematoma (**A-** *c–f*). 3D CT images showed the morphology of recurrent cerebral haemorrhage (**B-** *a–f*). **B** Expansile ventricles

**Fig. 2:**
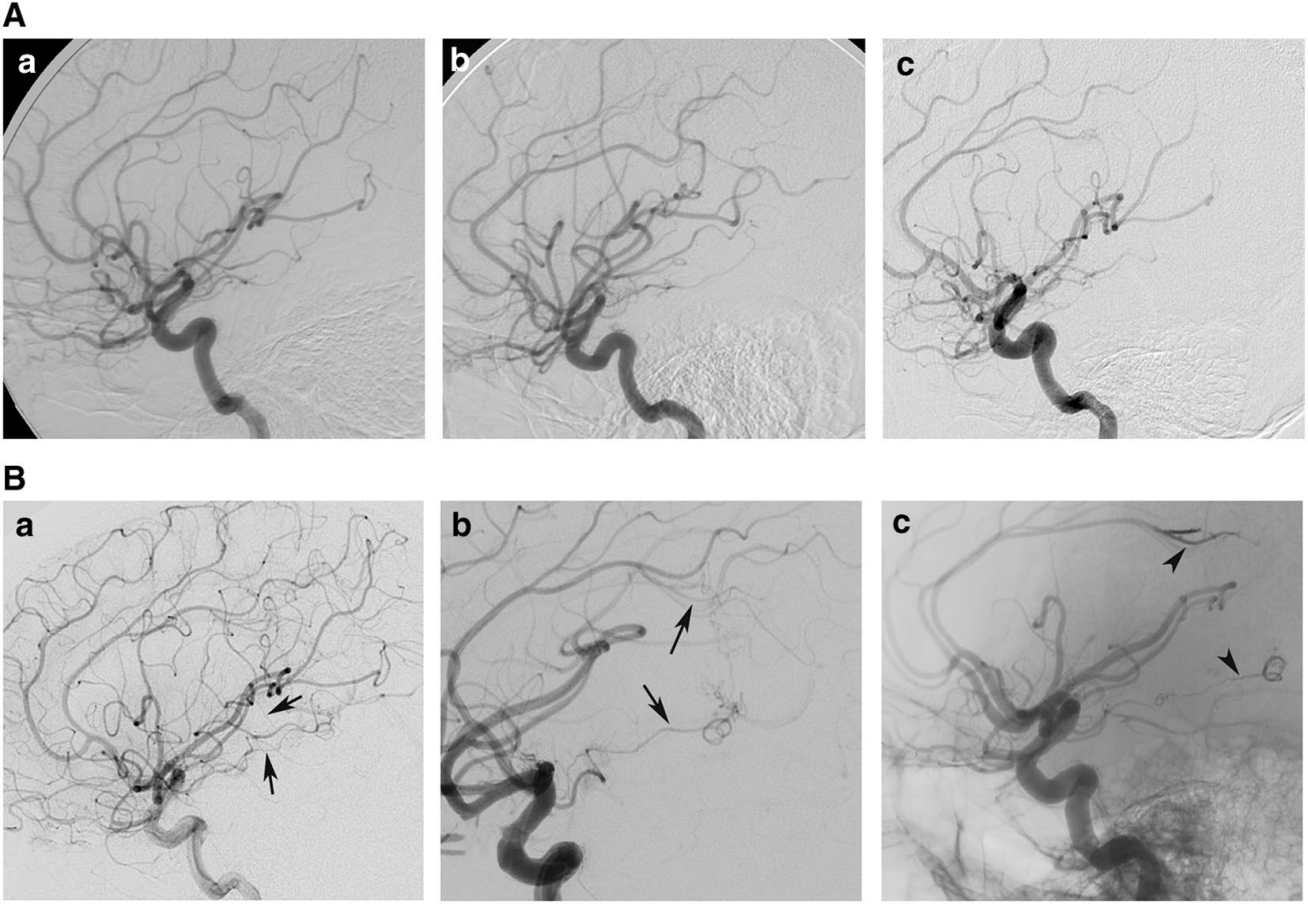
DSA images displayed the left ICA angioarchitecture from various examinations. Two micro AVMs, which were identified in the last examination (**B-** *a–c*), were not diagnosed in the first three DSA examinations (**A-** *a–c*). *Arrows* pointed at the feeding arteries of AVMs before embolization (**B-** *a, b*); and *arrowheads* showed the embolised feeding arteries after embolization (**B-** *c*)

A 21-year-old male patient was admitted to our center with acute headache and loss of consciousness in 2009. Intraventricular haematoma was confirmed by computed tomography (CT) scan (Fig. 1A-a, b). The patient recovered consciousness after ventricular drainage and the removal of intraventricular haematoma (Fig. 1A-c–f). One week after the haemorrhage, the patient was diagnosed with digital subtraction angiography (DSA) for the causes of haemorrhage, but no positive results were obtained (Fig. 2A-a). DSA was repeated 1 month later with a micro-catheter to obtain super-selective angiograpy of micro cerebral arteries, including posterior choroidal arteries. However, no cerebrovascular disease was identified (Fig. 2A-b; Additional file 1). The patient was discharged 1 month after his haemorrhage without any measurable neurological deficit.

In 2015, the patient was hospitalized in our center again for haemorrhage in splenium corporis callosi (Fig. 1B). He had headache, lost consciousness, and experienced plegia and aphasia. DSA was performed 3 days after haemorrhage. No sign of abnormal cerebral angioarchitecture was observed (Fig. 2A-c). Three months later, the patient was examined with DSA again after the cerebral haemorrhage was assimilated. A micro AVM with two non-compact niduses in different parts of the brain was identified. One of the niduses was supplied by an arteriole of the left callosomarginal artery and the other by a branch of the left anterior choroidal artery. These two micro niduses were drained via the same veinlet, one of the thalamic veins, to the great cerebral vein (Fig. 2B-a, b). The feeding arteries of these two niduses were embolised with 0.5 ml Onyx (Covidien, Irvine, CA, USA; Fig. 2B-c). In the end, the patient was discharged without any neurological deficit.

## Discussion

The annual haemorrhage incidence rate of multiple AVMs was 6.7 %, higher than that of the overall haemorrhage (Boone et al. 2016). In this case, two micro AVMs were diagnosed in different parts of the brain with common drainage in the dorsal thalamus (Fig. 2B-a, b). With the CT images of the haemorrhage morphologies (Figs. 1A-a, b, 2B), we believed that the lower AVM was associated with the first haemorrhage while the upper AVM was associated with the second haemorrhage. The incidence of AVM rupture depends on different risk factors, such as locations, volumes of nidus, AVM-associated aneurysms, sex, treatment modalities and draining veins (da Costa et al. 2009; Gross and Du 2013; Rutledge et al. 2014). It was reported that micro AVMs with low-shunting had higher pressure in feeding arteries and greater incidence of haemorrhage (Brown et al. 1988; Kader et al. 1994), consistent with our finding that the micro AVM with two diffuse and spread niduses drained into the same vein. Therefore, micro AVMs located in the deep brain should be detected and removed.

Before the identification of this micro AVM, we obtained negative results in all three previous DSAs. Spontaneous obliteration of AVMs after haemorrhages likely played an important role in those negative DSA results (Goyal et al. 2015). Small size niduses with single draining vein were mostly apt to have spontaneous thrombosis (Abdulrauf et al. 1999). At the same time, the haematoma and its compressive effects on the draining veins aggravated the spontaneous obliteration (Abdulrauf et al. 1999; Krapf et al. 2001). In this study, spontaneous obliteration of AVMs could be re-canalized and should have been identified. However, early angiography at the setting of haematoma might hinder the diagnosis of micro AVMs (Alen et al. 2013). Therefore both a proper diagnosis time and a regular follow-up are needed to detect those dormant AVMs. We have suggested this patient to have DSA every three to 6 months after first cerebral haemorrhage. Unfortunately, he did not follow our suggestion. It’s reported that some AVMs with spontaneous thrombosis were recanalized within 1 month to 5 years (Abdulrauf et al. 1999). Since we reported only one case here, it’s hard to draw a conclusion regarding the timing of the follow-up DSA. More cases are required in future studies to get a firm conclusion. Recently, the patient followed our suggestion and took the follow-up DSA after AVM embolization. No recanalization or de novo AVM was diagnosed (Additional file 2).

Younger patients did have re-haemorrhage and AVMs recurred in the same region (Ali et al. 2003). It remained unknown why the micro AVM ruptured at different locations over years for the patient in this study. AVMs were traditionally regarded as congenial lesions but they could also be de novo (Bulsara et al. 2002). In addition, AVMs can change over time. For example, angiogenic factors and inflammatory factors can activate or de-activate AVMs respectively (Kim et al. 2009). AVM patient with spread nidus and haemorrhage in different locations over years as reported in this study was rare and more studies at the molecular level are needed to understand the mechanism in the future.

## Conclusions

In summary, some micro, dormant, re-haemorrhagic AVMs located in deep brain can and should be diagnosed via DSA at an early time.

## Additional files

10.1186/s40064-016-2615-5 DSA images exhibited angiography of posterior cerebral circulation and micro-angiography of posterior choroidal arteries. Macro-angiographies of posterior circulation were displayed in panels **a** and **b**. Axial (**c**,**e**) and sagittal (**d**,**f**) views of micro-angiographies in left (**c**,**d**) and right (**e**,**f**) posterior choroidal arteries showed no abnormal angioarchitecture.
10.1186/s40064-016-2615-5 DSA images displayed cerebral angiography changes after AVM embolization. The images were taken 5 months after interventional embolization during the patient’s follow-up examination. No re-canalization or de novo AVM was detected.
