# Supplementary figures and images for: Dormant micro arteriovenous malformations lead to recurrent cerebral haemorrhage
Source: Springerplus. 2016 Jul 11;5(1):1054. doi: 10.1186/s40064-016-2615-5 (PMC4940323; doi:10.1186/s40064-016-2615-5)

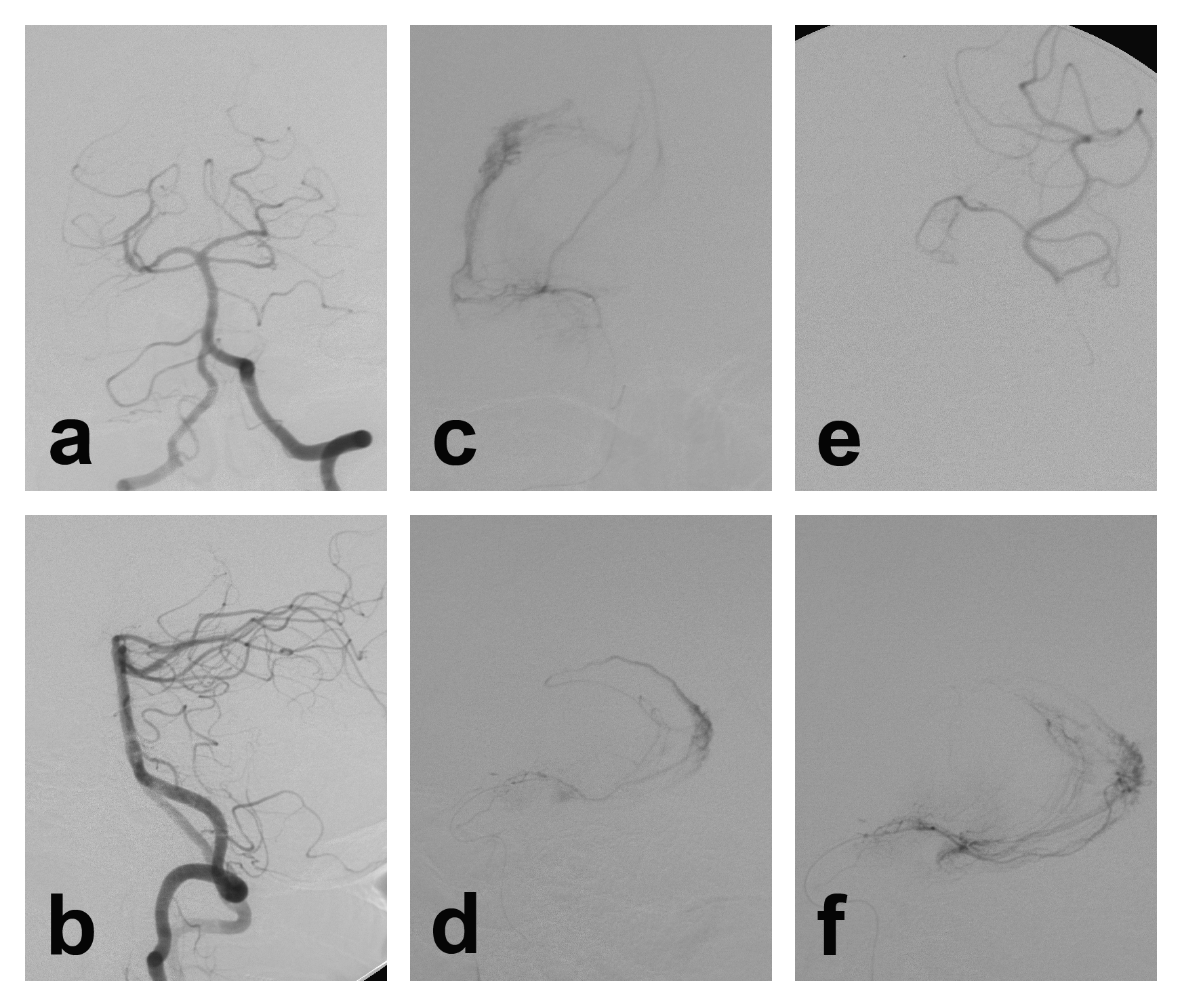

Supplement: Supplementary file 1 — 10.1186/s40064-016-2615-5 DSA images exhibited angiography of posterior cerebral circulation and micro-angiography of posterior choroidal arteries. Macro-angiographies of posterior circulation were displayed in panels a and b. Axial (c,e) and sagittal (d,f) views of micro-angiographies in left (c,d) and right (e,f) posterior choroidal arteries showed no abnormal angioarchitecture. [file 40064_2016_2615_MOESM1_ESM.tif]

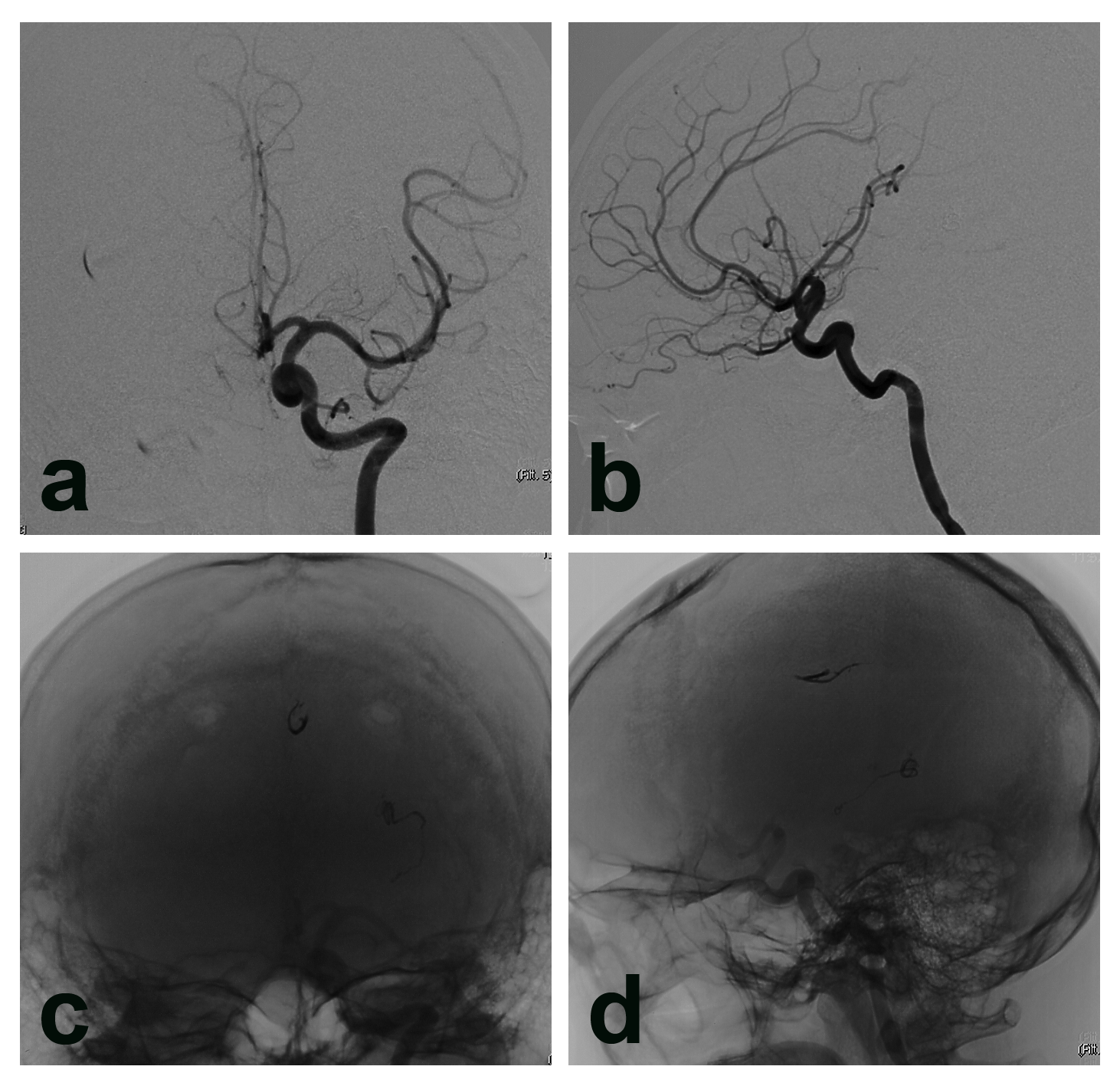

Supplement: Supplementary file 2 — 10.1186/s40064-016-2615-5 DSA images displayed cerebral angiography changes after AVM embolization. The images were taken 5 months after interventional embolization during the patient’s follow-up examination. No re-canalization or de novo AVM was detected. [file 40064_2016_2615_MOESM2_ESM.tif]
